# Symptoms and Quality of Life Changes after Hysteroscopic Treatment in Patients with Symptomatic Isthmocele—Preliminary Results

**DOI:** 10.3390/jcm10132928

**Published:** 2021-06-30

**Authors:** Monika Szafarowska, Magdalena Biela, Joanna Wichowska, Kamil Sobociński, Agnieszka Segiet-Święcicka, Jacek Doniec, Paweł Kamiński

**Affiliations:** 1 Department of Gynecology and Oncological Gynecology, Military Institute of Medicine, Szaserów 128 Street, 04-141 Warsaw, Poland; monika.szafarowska@wp.pl (M.S.); jrutkowska@wim.mil.pl (J.W.); ksobocinski@wim.mil.pl (K.S.); pkaminski@wim.mil.pl (P.K.); 2 Department of Experimental and Clinical Physiology, Medical University of Warsaw, Żwirki I Wigury 61 Street, 02-091 Warsaw, Poland; agnieszkasegiet@gmail.com; 3 Robotic Surgery Center, Military Institute of Medicine, Szaserów 128 Street, 04-141 Warsaw, Poland; jdoniec@wim.mil.pl

**Keywords:** hysteroscopy, niche, isthmocele, quality of life (QoL), abnormal uterine bleeding (AUB), sexual activity

## Abstract

Due to an increasing number of cesarean section deliveries, the common consequences of that surgery are observed more often in the population. One of them is the uterine cesarean scar defect known as niche or isthmocele. Most patients with that aliment are asymptomatic, but some of them can report abnormal uterine bleeding, pelvic pain, subfertility which can be the reason for reduced quality of life (QoL) of the patients. In our study, we analyzed the subjective feelings of changes in the severity of symptoms and quality of life of women with niche after diagnostic and operative hysteroscopy. The patients *n* = 85 included in the study group completed a follow up questionnaire six months after the procedure. Patients after operative hysteroscopy in comparison to patients after diagnostic procedure reported statistically significant reduction in post-menstruation bleeding/spotting and improvement in the quality of sexual activity. We have also noticed a higher pregnancy rate in the operative group, however, the difference was not statistically significant. According to our study, most women reported a positive effect of hysteroscopy on their QoL in social, psychological, environmental, and health domains.

## 1. Introduction

Isthmocoele, also known as “niche”, is a common problem in gynecological and obstetrical care and it develops in 50–60% of women who have had a cesarean section [1]. As the number of cesarean section deliveries grows, cesarean scar defects and its implications must be well diagnosed and properly treated. Treatment of symptomatic isthmocele mainly depends on individual fertility desires. The wide range of therapeutic options allows the gynecologist to choose the personalized form of therapy. For the patients without reproductive plans, the treatment may consist of oral contraception, hormonal intrauterine device, more or less invasive operations such as hysteroscopy, laparoscopy, or even hysterectomy [2]. However, for patients with fertility problems, the treatment depends on residual myometrium thickness (RMT) assessed in transvaginal ultrasound examination. When RMT ≥2.5 mm, the hysteroscopic resection can be performed. In case the RMT is less than <2.5 mm, laparoscopy, laparotomy, or vaginal approach repair is recommended [3].

It is widely understood that isthmocele has a negative impact on patients’ quality of life. According to Stegwee et al., the most relevant symptoms for QoL in isthmocele patients were abnormal uterine bleeding (AUB), subfertility, sexual activity, and self-esteem [1]. When it comes to AUB, quality of life is decreased because of AUB-associated odor, recurrent fungal infections, irregularity of AUB, discomfort during leisure activities (e.g., sport), and a lower work capacity. Furthermore, AUB may directly lead to an inability to have sexual relations. Fear of bleeding or spotting during intercourse may cause deep embarrassment during intercourse or avoiding intercourse altogether. Due to bleeding, the libido or lubrication may be decreased, accompanied with a fear of disappointing a partner. Sexual activity and self-esteem seem to be one of the most important factors that impact the quality of life of niche patients. 

The aim of our study was to examine the improvement of QoL in social, psychological, environmental, and health domains in niche-related patients after diagnostic and operative hysteroscopy. 

## 2. Materials and Methods

A prospective study was conducted at the Department of Gynecology and Oncological Gynecology of the Military Institute of Medicine, Warsaw, Poland, between 2017 and 2020. The study was approved by the institutional review board and supported by the Statutory Grant No. 433 of the Military Institute of Medicine, Warsaw, Poland. Written informed consent was obtained from all patients. Each of the participants was informed about the purpose of this study.

**Figure 1:**
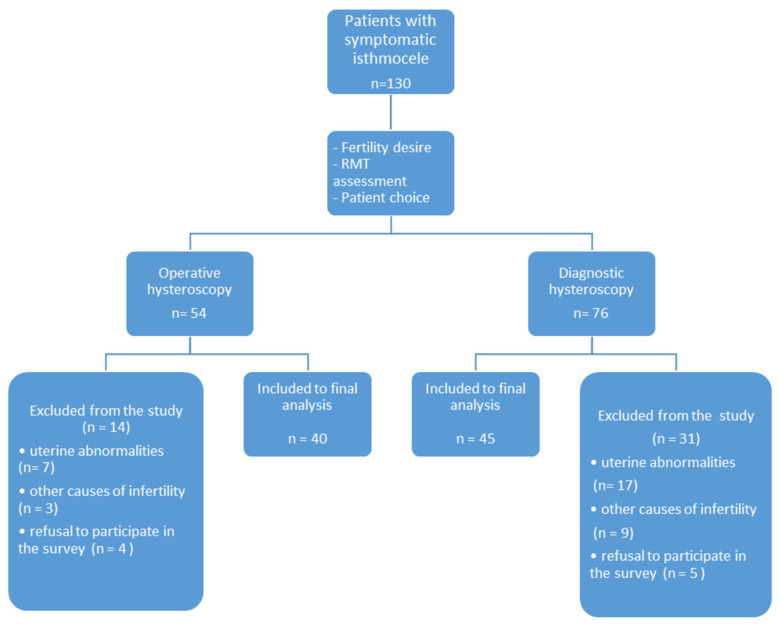
The scheme of the study. RMT: residual myometrium thickness.

One hundred and thirty patients with symptomatic isthmocele (*n* = 54 after operative, and *n* = 76 after diagnostic hysteroscopy) were evaluated. However, only eighty-five patients aged 24–49 were finally included in the study group (*n* = 40 after operative, and *n* = 45 after diagnostic hysteroscopy) because of the exclusion criteria. The initial diagnosis of isthmocele was based on a transvaginal ultrasonography (TVUS) and clinical signs. Transvaginal ultrasound scans were performed in all patients in the proliferative phase to access the isthmocele according to Delphi protocol [4]. Patient demographics and physical examination data were obtained from the medical documentation of patients and patient surveys carried out for that purpose. Every patient was informed about the possibility of performing a diagnostic or operative procedure and all possible benefits and complications were discussed. The decision about the planned surgery was made after the hospital admission and depended on the transvaginal ultrasound result. All women with residual myometrial thickness (RMT) <2.5 mm assessed on TVUS were qualified for the diagnostic mini-hysteroscopy, whereas the women with RMT ≥2.5 mm were qualified for the hysteroscopic isthmocele repair. The scheme of the study is presented in Figure 1.

In each patient, a hysteroscopy was performed in the proliferative phase of the menstrual cycle. The rigid hysteroscope, Karl Storz Endoscope, Germany, with an oval profile and a width of 4 mm mini-hysteroscope or a 5 mm office mini-resectoscope, was used for all patients. During the hysteroscopy, the distal edge of the defect, the size of the isthmocele, abnormalities within the defect (abnormal vessels, polyps), the proximal edge, and the endometrium in the uterine cavity were assessed. 

The diagnostic mini-hysteroscopy was performed following aseptic rules, according to prof. S. Bettocchi’s method [5]. During this procedure, the residual blood and mucus within the niche was removed. In the case of women with abnormal uterine bleeding, the endometrial biopsy was performed. 

The hysteroscopic isthmocele repair procedure was performed with a 5 mm mini-resectoscope, under general anesthesia, in accordance to the protocol including resection of the distal part of the edge, creating a 2–3 mm wide canal in the proximal part of the anterior cervical lip, facilitating the outflow of residual isthmocele secretions, and coagulation of abnormalities within the isthmocele. In the case of women with abnormal uterine bleeding, the endometrial biopsy was performed.

Six months after the hysteroscopy, a follow up questionnaire was performed. All patients provided information about the result of the procedure, assessed the impact of the procedure on improving their quality of life in the physical, social, environmental, and psychological domains. The survey was prepared by the authors based on the research of the Stegwee et al. [1] and included simple questions about subjective feelings related to the niche symptoms influencing QoL. From the final analysis, we excluded all patients in whom other pathologies such as: polyp, myoma, or congenital abnormalities were detected during hysteroscopy. Moreover, women with other known causes of reproductive failure were also excluded from the study group.

### Statistical Analysis

In descriptive statistics for categorical variables, the number and percentage of occurrences were reported. The distribution of continuous variables was first evaluated with the Shapiro–Wilk test, then, in descriptive statistics for normally distributed variables, mean with standard deviation (SD) and range (min-max) were reported, otherwise median with the 25th and 75th percentile (Q1 and Q3) and range (min-max) were provided. Categorical variables were compared using the Fisher test or Chi-squared test, depending on the size of the categories. The normally distributed continuous variables were compared using the Student’s t-test, otherwise the Mann–Whitney test was used.

To assess the effect of hysteroscopy on the possibility for improvement in each symptom after the procedure, univariable logistic regression models were used. The significance level was set at 0.05. Two-sided tests were used. Analysis was performed with R statistical software, version 3.6.3 (R Core Team (2020). R: A language and environment for statistical computing. R Foundation for Statistical Computing, Vienna, Austria. URL https://www.R-project.org/, accessed on 25 May 2021).

## 3. Results

### 3.1. Characteristics of the Study Population

After a detailed analysis, we included *n* = 85 patients in the study, who declared a deterioration in the quality of life due to isthmocele symptoms. Forty-one patients, *n* = 41 (48.2%), included in our study reported three and more symptoms, *n* = 27 (31.8%) patients reported two symptoms, and *n* = 17 (20%) patients reported only one symptom of the isthmocele.

**Figure 2:**
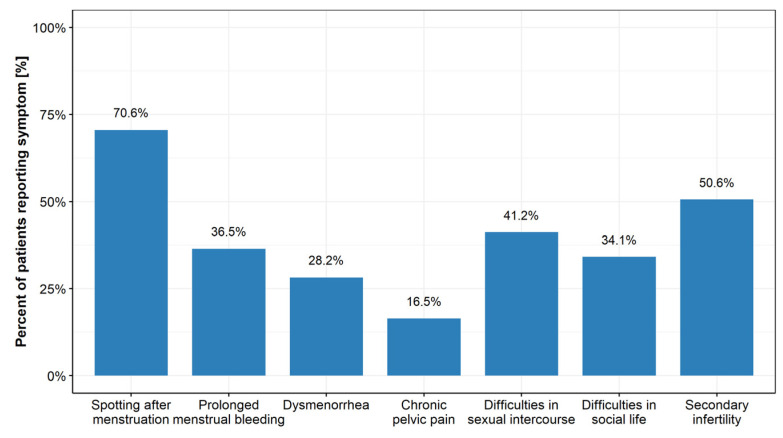
The most common symptoms of the isthmocele reported by patients.

The most common complaints reported by the patients are presented in Figure 2.

All women included in our study had at least one previous cesarean section, *n* = 50 women (58.8%) had one cesarean section, *n* = 29 women (34.1%) had two cesarean sections, *n* = 4 women (4.7%) had three previous cesarean sections, and *n* = 2 (2.4%) had four cesarean sections. Regarding the anatomical position of the uterus, *n* = 59 (69%) women presented anteverted and *n* = 26 (31%) presented retroverted uterus. The results of our study were divided according to the main QoL domain and consist of physical, psychological, social, and environmental factors.

#### 3.1.1. Physical Health

Assessment of the type of hysteroscopy treatment and their correlation with the improvement of symptoms influenced QoL in physical health domains are presented in Table 1 and Table 2.

According to the presented data, most women reported a positive effect of the hysteroscopy on improving the QoL. However, the logistic regression analysis showed a significantly higher effectiveness of operative hysteroscopy as compared to diagnostic hysteroscopy in the reduction of symptoms associated with postmenstrual spotting OR (95% CI) 3.437 (1.134–11.488), *p*-value 0.035. In the case of heavy bleeding, painful periods, or chronic pain, no significant differences were observed depending on the type of procedure performed.

#### 3.1.2. Subfertility

The analysis of the group of infertility patients showed that 52% of women who underwent operative hysteroscopy became pregnant compared to 26% of women who underwent diagnostic hysteroscopy, but the result is not statistically significant (Table 2). Logistic regression analysis showed no relationship between the type of hysteroscopy performed and pregnancy outcome OR (95% CI) 3.033 (0.870–11.815); *p*-value 0.091. Most of the pregnancies (68.4%) were spontaneous and IVF procedure was performed in 31.6% of women.

#### 3.1.3. Psychological and Environmental Aspect

Twenty-nine (34%) of the women included in the study confirmed the presence of symptoms lowering the quality of life in terms of psychological and environmental domain. The main symptoms reported by the patients on the psychological and environmental levels, as well as the effect of the treatment and thus improvement of the quality of life, are presented in Table 3.

#### 3.1.4. Social Health

Among the symptoms analyzed in terms of the deterioration of the quality of life on the social health domain, sexual intercourse seems to be the most important. As many as 41.2% of women reported a deterioration in the quality of intercourse, and indicated spotting after intercourse as the main problem (80%). The performed hysteroscopy reduced the symptoms associated with sexual intercourse, and thus improved the quality of life in 66.7% of women. It is very important to emphasize that the relief of symptoms was declared by 93.3% of women after operative hysteroscopy vs. 44.4% of women after diagnostic hysteroscopy (*p* = 0.009). Moreover, the performed logistic regression analysis showed a significantly higher effectiveness of operative hysteroscopy compared to diagnostic hysteroscopy in the reduction of symptoms associated with spotting after sexual intercourse OR ratio (95% CI) 16.500 (2.301–344.405), *p*-value 0.017. The analysis of the effect of the hysteroscopic treatment and thus the improvement of the quality of life is presented in Table 4.

## 4. Discussion

Nowadays, about 20% of pregnant women undergo cesarean section delivery worldwide [6]. According to the National Health Fund (NFZ), in 2018, the percentage of cesarean sections in Poland was even higher, about 43.85% [7]. Because of that, the side effects of this procedure can be observed more often. The cesarean scar defect, which can be also known as: cesarean scar dehiscence, isthmocele, uterine transmural hernia, diverticulum, pouch formation, niche, uteroperitoneal fistula is one of the most common consequences of this technique [8]. Etiology, risk factors, and the treatment methods of the niche are under continuous evaluation. However, there is lack of research investigating the impact of the cesarean scar defect symptoms on women’s quality of life (QoL), and effects of the hysteroscopic treatment on the changes in QoL. Based on the conclusions of the study of Stegwee et al. [1], we evaluated the most frequent niche symptoms and their impact on the four aspects of life that were most prioritized by women. Moreover, we have assessed changes observed by patients about six months after a diagnostic or operative hysteroscopy to find out the impact of the minimally invasive treatment on the women’s QoL.

According to our findings, hysteroscopy in general has a positive effect on the symptoms and the QoL of women with the cesarean scar defect. The most significant improvement was observed in the duration of the postmenstrual spotting after the operative hysteroscopy procedures compared to the diagnostic procedures. After the operative hysteroscopy, about 79% of women reported improvement of those symptoms, compared to 52% of women after diagnostic procedure. Similar conclusions were presented by Muzzi et al. in the prospective randomized study of 47 patients. Investigators observed a significantly shortening of the duration of periods compared with the duration of menses before the operative hysteroscopy in the treated group [9]. Moreover, according to the recent literature review, hysteroscopic remodeling relieved symptoms of AUB in 60% to 100% of women with a niche. The improvement of the symptoms is comparable after hysteroscopic and laparoscopic procedures (60–100% vs. 78–94%) [10]. It is important to highlight that, the hysteroscopic resection of isthmocele is a minimally invasive, non-time consuming, and low morbidity procedure, in comparison to the laparoscopic surgeries [11]. The other important feature of hysteroscopy is the diagnostic value of other possible reasons for AUB. In our study, 24 patients that underwent a hysteroscopy (seven in the operative group and 17 in the diagnostic group) had additional findings according to the PALM-COEIN classification. Those pathologies might increase the severity of symptoms or, in some situations, they could be the only reason for its occurrence. Performing the correction of the niche via laparoscopic or vaginal approach without previous hysteroscopic evaluation of the uterine cavity could result in underdiagnosing of the patient [2]

Abnormal uterine bleeding and spotting between menses or after intercourse are the possible reasons for the lower quality of life in patients with isthmocele. Those symptoms negatively affect many aspects of life also including sexual activity. Prolonged, unpredictable bleeding can cause aversion to sexual activity, decreased libido, and lubrication but also can provoke the fear of disappointing the partner and a sense of embarrassment due to a lower self-esteem [1]. That problem might be even more significant in specific cultures or religions, where women during vaginal bleeding are impure and unclean, and therefore, spotting women are isolated, prohibited from polluting the holy places, and shunned [12]. They also could not have intercourse until the bleeding stopped. In our study group, about 41.2% of patients reported a lower quality of sexual life caused by the niche-related symptoms. However, it is comforting that minimal invasive treatment has a positive effect on that aspect of life, as it is clearly shown in the results. The operative hysteroscopy successfully reduced the postcoital spotting in more than 91% of women in comparison to only 40% of women after a diagnostic hysteroscopy, and the results are statistically significant. Moreover, the hysteroscopic surgery improved dyspareunia and reduced the unpleasant smell of the vagina. Our results are similar to other researchers [9,11], but further investigations are needed.

All of our patients were properly informed about their condition at the beginning of the study. We believe that a well informed patient feels more safe and confident about the proposed treatment. In our research, special interest was on patients of reproductive age, especially those with a subfertility. In that group, we put special attention to inform patients that hysteroscopy usually does not result in thickening of the remaining myometrium. All of our patients were also aware of the risk of niche dehiscence in pregnancy or during delivery. In our study, we refer to the operative hysteroscopy only women with RMT 2.5 mm or more, even though some authors suggest that in the case of reproductive plans, only patients with RMT more than 3.5 mm should be qualified for hysteroscopic resection [13]. If the RMT was thicker, we conducted a diagnostic hysteroscopy and eventually referred patients for a laparoscopic repair. It is worth noting that only in a few studies, the RMT was evaluated after hysteroscopic resection. Results suggest a slight thickening or unchanged thickness of RMT after the surgery [14,15]. In our study, we did not check that parameter, but it could be an interesting subject for further research.

There are many explanations for the hypothesis of subfertility caused by an isthmocele such as: endometrial fluid remaining in niche, when the isthmocele acts as a “third sactosalpinx” or a subclinical inflammation of endometrium [16]. Regardless of the cause, it is confirmed that the live birth rate is lower in patients after cesarean section delivery than in women with a previous vaginal delivery and the difference is even more prominent in patients with confirmed niche. Visser et al. reported the life birth rate at 10.7% after ET in women with isthmocele in comparison to 23.3% in women with only vaginal delivery history [17]. According to our results, about 52% of patients after operative hysteroscopy and 26% after diagnostic hysteroscopy got pregnant. The fact is worth noting, that in this group, 68.4% of pregnancies were spontaneous. Our results do not meet the statistically significance level, but the difference between those groups is clearly seen. Moreover, none of our patients reported a spontaneous miscarriage or an ectopic pregnancy in the cesarean section scar. In the literature, the pregnancy rate after hysteroscopic resection ranged from 6.6% up to 100% [18].

## 5. Conclusions

Hysteroscopy seems to have a positive effect on the QoL of patients with isthmocele. Specifically, hysteroscopic resection of the niche could increase sex life quality and reduce the postmenstrual spotting. Moreover, operative hysteroscopy could have a positive effect on niche-related subfertility, but further research is needed. 

## Figures and Tables

**Table 1:** The diagnostic vs. operative hysteroscopy and their influence on the reported symptoms.

		Hysteroscopy			
Characteristic	N	Overall, N = 85 ^1^	Diagnostic Hysteroscopy, N = 45 ^1^	Operative Hysteroscopy, N = 40 ^1^	*p*-Value ^2^
**Prolonged menses**	31				0.697
improvement after hysteroscopy		21 (67.7%)	12 (63.2%)	9 (75.0%)	
unsuccessful treatment		10 (32.3%)	7 (36.8%)	3 (25.0%)	
**Postmenstrual spotting**	59				**0.018**
improvement after hysteroscopy		38 (64.4%)	16 (51.6%)	22 (78.6%)	
unsuccessful treatment		21 (35.6%)	15 (48.4%)	6 (21.4%)	
**Dysmenorrhoea**	24				>0.999
improvement after hysteroscopy		12 (50.0%)	6 (46.2%)	6 (54.5%)	
unsuccessful treatment		12 (50.0%)	7 (53.8%)	5 (45.5%)	
**Pelvic pain**	13				>0.999
improvement after hysteroscopy		9 (69.2%)	5 (71.4%)	4 (66.7%)	
unsuccessful treatment		4 (30.8%)	2 (28.6%)	2 (33.3%)	

^1^*n* (%); ^2^ Fisher’s exact test; Pearson’s Chi-squared test.

**Table 2:** The diagnostic vs. operative hysteroscopy and their influence on pregnancy outcomes.

Characteristic	N = 44 ^1^	Diagnostic Hysteroscopy	Operative Hysteroscooy	*p*-Value ^2^
**Pregnancy after hysteroscopy**				0.159
YES	18 (40.9%)	5 (26.3%)	13 (52.0%)	
NO	26 (59.1%)	14 (73.7%)	12 (48.0%)	

^1^*n* (%); ^2^ Pearson’s Chi-squared test; Fisher’s exact test.

**Table 3:** The diagnostic vs. operative hysteroscopy and their influence on the psychological and environmental domain.

Characteristic	N	Overall ^1^	Diagnostic Hysteroscopy ^1^	Operative Hysteroscopy ^1^	*p*-Value ^2^
**Avoiding participation in leisure activities**	14				0.266
improvement after hysteroscopy		9 (64.3%)	3 (42.9%)	6 (85.7%)	
unsuccessful treatment		5 (35.7%)	4 (57.1%)	1 (14.3%)	
**Necessity to have a toilet nearby**	9				0.083
improvement after hysteroscopy		3 (33.3%)	1 (14.3%)	2 (100.0%)	
unsuccessful treatment		6 (66.7%)	6 (85.7%)	0 (0.0%)	
**Limited working capacity**	4				
improvement after hysteroscopy		3 (75.0%)	3 (75.0%)	0 (NA%)	NS
unsuccessful treatment		1 (25.0%)	1 (25.0%)	0 (NA%)	
**Low self esteem**	3				>0.999
improvement after hysteroscopy		2 (66.7%)	1 (50.0%)	1 (100.0%)	
unsuccessful treatment		1 (33.3%)	1 (50.0%)	0 (0.0%)	
**Loneliness or depression**	11				0.061
improvement after hysteroscopy		5 (45.5%)	0 (0.0%)	5 (71.4%)	
unsuccessful treatment		6 (54.5%)	4 (100.0%)	2 (28.6%)	

^1^*n* (%) ^2^ Fisher’s exact test.

**Table 4:** The diagnostic vs. operative hysteroscopy and their influence on sexual activity.

Characteristic	N	Overall, N = 11 ^1^	Diagnostic Hysteroscopy, N = 7 ^1^	Operative hysteroscopy, N = 4 ^1^	*p*-Value ^2^
**Dyspareunia**	10				>0.999
improvement after hysteroscopy		7 (70.0%)	5 (71.4%)	2 (66.7%)	
unsuccessful treatment		3 (30.0%)	2 (28.6%)	1 (33.3%)	
**Spotting after intercourse**	27				**0.014**
improvement after hysteroscopy		17 (63.0%)	6 (40.0%)	11 (91.7%)	
unsuccessful treatment		10 (37.0%)	9 (60.0%)	1 (8.3%)	
**Unpleasant odor**	8				>0.999
improvement after hysteroscopy		4 (50.0%)	2 (40.0%)	2 (66.7%)	
unsuccessful treatment		4 (50.0%)	3 (60.0%)	1 (33.3%)	
**Reduced lubrication**	3				>0.999
improvement after hysteroscopy		1 (33.3%)	0 (0.0%)	1 (50.0%)	
unsuccessful treatment		2 (66.7%)	1 (100.0%)	1 (50.0%)	
**Reduced libido**	3				NS
improvement after hysteroscopy		2 (66.7%)	0 (NA%)	2 (66.7%)	
unsuccessful treatment		1 (33.3%)	0 (NA%)	1 (33.3%)	

^1^*n* (%) ^2^ Fisher’s exact test.

## Data Availability

The datasets generated and analyzed during the current study are not publicly accessible but are available from the main author on reasonable request.
